# Supplementation with mulberry leaves improves growth performance and meat quality of Xiangdong black goats

**DOI:** 10.5713/ab.24.0427

**Published:** 2024-08-26

**Authors:** Yang Luo, Shuai Gao, Ao Sun, Jianbo Li, Haobang Li, Kangle Yi, Renke Hu, Bin Yang

**Affiliations:** 1 Key Laboratory of Molecular Animal Nutrition, Ministry of Education, Zhejiang University, Hangzhou 310058, China; 2 College of Animal Science and Technology, Guangxi University, Nanning 530004, China; 3 Hunan Institute of Animal and Veterinary Science, Changsha 410130, China; 4 School of Biological and Chemical Engineering, Zhejiang University of Science and Technology, Hangzhou 310023, China

**Keywords:** Antioxidant Activity, Growth Performance, Meat Quality, Mulberry Leaf, Xiangdong Black Goat

## Abstract

**Objective:**

Mulberry (*Morus alba*) leaf (ML) is a high-quality feed source for ruminants, while it is unclear whether it can enhance the growth performance and meat quality of Xiangdong black goats.

**Methods:**

In this study, we investigated the effects of ML supplementation (0%, 5%, 10%, 15%, and 20%) on the growth performance, serum variables, and the profiles of amino acids and fatty acids in the muscle of Xiangdong black goats.

**Results:**

Results showed that the final body weight, initial and final dry matter intake, and average daily gain increased linearly and quadratically with the increasing ML content (p<0.05). The serum concentrations of total antioxidant capacity (T-AOC) increased linearly, while immunoglobulin G (IgG) increased quadratically with the increasing ML content (p<0.05). Conversely, the saturated fatty acids (SFA) content in meat decreased linearly with the increasing ML content (p<0.05). Compared to goats without ML supplementation, goats fed with 15% ML showed significant increases in serum concentrations of T-AOC, superoxide dismutase, catalase, and IgG (p<0.05). Furthermore, goats fed with 20% ML displayed significant decreases in SFA (C18:0) content, compared to goats without ML supplementation (p<0.05).

**Conclusion:**

These results suggest that ML supplementation promotes the growth performance of goats. A diet containing 15% ML showed better effects in promoting antioxidant and immunomodulatory activities, while a diet with 20% ML was more effective in enhancing meat flavor in Xiangdong black goats.

## INTRODUCTION

The mulberry (*Morus alba*) leaf (ML) is an important product of mulberry, which has a significant production in China [1]. It is commonly used as a Chinese traditional medicine for humans and as a food source for silkworms. Since MLs have a high protein content with good essential amino acid profiles, low content of tannin and phytic acid content, and combine properties of good palatability and high digestibility for herbivores, they are gradually being introduced as livestock feed in recent years [2]. In addition, ML contain abundant bioactive compounds such as polysaccharides, flavonoids, phenolic acids, and alkaloids, which have antioxidant, antibacterial, and immunomodulatory properties [3–5]. The use of ML as feed for livestock not only provides animals with exceptional nutritional value and important biological activity but also helps utilize surplus mulberry resources efficiently.

Studies have been conducted to investigate the specific effects of ML on various ruminants. Ouyang et al [6] found that supplementing the concentrate diet with 30% ML powder could enhance nutrient digestibility and support the development of rumen epithelium in 3 mo fattening Hu sheep without affecting growth performance. Sun et al [7] suggested adding 24% ML to the diets as it increased intramuscular fat and monounsaturated fatty acid (MUFA) levels while reducing saturated fatty acid (SFA) content in the muscle of 4 mo small-tailed Han sheep. Long et al [8] demonstrated that a 40% ML supplementation had the most significant effects on improving growth performance and apparent digestibility, promoting the deposition of intramuscular fat and inosinic acid, and enhancing the profile of fatty acids and amino acids in the muscles of 5 to 6 mo Guizhou crossbred black goats. These findings indicate that although ML offer beneficial effects on growth performance and muscle quality, the optimal supplementation ratio of ML varies for each type of ruminant.

The Xiangdong black goat (*Capra hircus*) is an outstanding local goat breed known for its black hairy coat. These goats are well-suited to challenging environments and rougher feed. Moreover, their meat is characterized by low odor, high lean content, delicious flavor, and exceptional nutritional value [9,10]. We hypothesized that supplementing with ML would enhance the growth performance and meat quality of Xiangdong black goats. Therefore, the objectives of this study were to investigate the effects of ML supplementation on the growth performance, serum variables, and the profiles of amino acids and fatty acids in the muscle of Xiangdong black goats.

## MATERIALS AND METHODS

### Animal care

The experiment was conducted at the experimental farm of the Hunan Institute of Animal and Veterinary Science. The protocol and experimental procedures used in this study were approved by the Institutional Animal Care and Use Committee (IACUC) of the Hunan Institute of Animal and Veterinary Science (HIAVS) (Approval No. HIAVS-IACUC-2024-03).

### Animals and experimental design

Twenty-five healthy male Xiangdong black goats (3 to 4 mo of age) with an average body weight (BW) of 13.17±1.67 kg (mean±standard error) were purchased from a commercial farm (Liuan Agricultural Science and Technology Comprehensive Development Co., Ltd, Liuyang, China). The goats were randomly divided into 5 groups (including Con, ML5, ML10, ML15, and ML20) and were fed with pelleted diets containing 0%, 5%, 10%, 15%, and 20% ML (air-dry basis), respectively, with 5 goats in each group. The ML used in this study were provided by Changsha Jinchang Silkworm Breeding Professional Cooperative (Changsha, China). The ML were air-dried and ground into powder to mix with other ingredients to make pelleted diets that were 1.5 cm long and 6 mm in diameter. The protein level of the ML powder was 14.8% of dry matter (DM). The ingredients and chemical composition of the diets are shown in Table 1. The contents of soybean meal and straw were modified to achieve similar levels of crude protein (CP) and metabolism energy (ME) among the different diets. The experimental goats were housed in 5 indoor pens (3.5 by 1.4 m) and were allowed to adapt to the feeding environment for 10 d. During the experimental period, the orts were removed once daily before the morning feeding, and new feed was delivered twice daily at 07:00 and 17:00. Five goats within one group shared one feed trough. All goats had free access to fresh water. The feeding experiment was conducted for 92 d.

### Sample collection and performance measurement

The feed intake of each group was recorded daily. The initial dry matter intake (DMI) and final DMI were calculated based on the mean DMI of the 7 d at the beginning and end of the feeding trial. The BW of goats was measured on d 0 and 92 of the feeding trial. On d 92, all goats were fasted for 24 h, then sacrificed by electrical stunning and exsanguination. Before sacrifice, 10 mL of blood was collected from the jugular vein of each goat into a heparinized vacutainer tube on d 92. Subsequently, the blood samples were centrifuged at 1,500×g at 4°C for 15 min to obtain the serum, which was then stored at −80°C until further analysis. The right-side longissimus dorsi muscle was collected immediately after sacrifice and stored at −20°C for further analysis.

### Chemical analysis

The DM (method 924.05), CP (method 988.05), ether extract (method 954.02), calcium (method 935.13), and phosphorus (method 964.06) of feed samples were determined according to AOAC Official Method [11]. Ash was determined according to a modified method of AOAC Official Method 942.05 [12]. The ME was determined using a calorimeter (Kaiyuan, Changsha, China). The neutral detergent fiber (with heat-stable amylase and sodium sulfite) and acid detergent fiber contents were determined using Ankom filter bags and fiber analyzer equipment (Ankom Technology, Macedon, NY, USA) [13].

### Serum analysis

The serum concentrations of glucose, total protein, blood urea nitrogen, cholesterol, triglycerides, high-density lipoprotein, low-density lipoprotein, and very low-density lipoprotein were determined using commercial kits (Nanjing Jiancheng Bioengineering Institute, Nanjing, China) on a Hitachi 7020 autobiochemistry instrument (Hitachi, Tokyo, Japan). The serum concentrations of immunoglobulin A (IgA), G (IgG), and M (IgM), as well as complement 3 (C3) and 4 (C4) were determined with commercial ELISA kits (mlbio, Shanghai, China) using a microplate reader (RT-6100; Rayto, Shenzhen, China). The serum concentrations of total antioxidant capacity (T-AOC), superoxide dismutase (SOD), catalase (CAT), and malondialdehyde (MDA) were determined using commercial kits (Beijing Huaying Biotechnology Research Institute, Beijing, China) on the microplate reader.

### Muscle amino acid analysis

The amino acid profile of each muscle sample was measured using an ion-exchange amino acid analyzer (L-8900; Hitachi, Japan) following AOAC (2000). Briefly, 0.1 g of dried and defatted muscle samples were mixed with 10 mL HCl (6 mol/L) and hydrolyzed at 110°C for 24 h. The hydrolysate was then diluted to 100 mL with distilled water. A 1-mL subsample was filtered through a 0.45-mm membrane and subsequently diluted tenfold for amino acid analysis.

### Muscle fatty acid analysis

The ground muscle sample (1 g) was methylated according to Sun et al [14]. The methyl esters were separated and quantified using a gas chromatograph (7890A GC System; Agilent, Santa Clara, CA, USA) equipped with a flame-ionization detector and a 30-m fused silica capillary column with 0.25 mm inner diameter and 0.25 mm film thickness (ECONO-CAP EC100 FFAP; Alltech, Bridgewater, NJ, USA). The gas chromatography program was initiated at 140°C for 4 min, then raised in 5°C/min increments to 230°C, and maintained at 230°C for 10 min. The temperature of the injector and detector was 280°C and 300°C, respectively. Nitrogen was used as a carrier gas at a flow rate of 30 mL/min. Peak identification was based on fatty acid methyl esters standards. Fatty acid compositions were determined from the chromatogram peak areas and expressed as g/100 g of identified total fatty acid methyl esters.

### Statistical analysis

The results were analyzed using a completely randomized study design. All experimental data were subjected to IBM SPSS Statistics 25 software (IBM Corp, Armonk, NY, USA). An orthogonal polynomial regression analysis was conducted to examine the linear (L) and quadratic (Q) effects of ML supplementation. Duncan’s multiple comparison test was performed on the mean values among different groups. Significance was defined as p<0.05, and a trend was defined as 0.05≤p<0.10.

## RESULTS

### Growth performance

As shown in Table 2, the initial BW of goats was similar among different groups, while the final BW (*P*_L_ = 0.014 and *P*_Q_ = 0.033), average daily gain (ADG; *P*_L_ = 0.003 and *P*_Q_ = 0.009), initial and final DMI (*P*_L_<0.001 and *P*_Q_<0.001) significantly increased with ML supplementation both linearly and quadratically. The initial and final DMI significantly increased in ML15 and ML20 when compared to Con, ML5, and ML10 (p<0.05). The ML20 goats had the highest ADG, and initial and final DMI, with values of 166.1 g/d, 1.11 kg, and 1.32 kg, respectively.

### Serum variables

Among the serum variables, the concentration of IgG increased quadratically with ML supplementation (*P_Q_* = 0.049; Table 3). Compared to Con, the concentration of IgG significantly increased in both ML10 and ML15 goats (p<0.05; Table 3). In addition, the concentration of glucose showed a linear tendency to increase with ML supplementation (*P_L_* = 0.063; Table 3). No significant changes were observed in other serum variables.

### Antioxidant properties

The serum concentration of T-AOC increased linearly (*P*_L_ = 0.018) and tended to increase quadratically (*P*_Q_ = 0.051) with the increasing ML supplementation (Table 4). The serum concentration of SOD tended to increase quadratically with the increasing ML supplementation (*P*_Q_ = 0.070; Table 4). Among the different groups, the ML15 goats had the highest concentrations of T-AOC, SOD, and CAT, while had the lowest concentration of MDA (Table 4).

### Amino acid and fatty acid profiles

The concentration of Tyr tended to increase linearly with ML supplementation (*P*_L_ = 0.087; Table 5). The SFA decreased linearly (*P*_L_ = 0.046) and tended to decrease quadratically (*P*_Q_ = 0.091) with increasing ML supplementation (Table 6). Compared to Con, the concentration of C18:0 was significantly decreased in ML20 goats (p<0.05; Table 6). No significant changes were observed in other amino acid or fatty acid concentrations.

## DISCUSSION

Due to the good palatability of ML with low fiber content but high CP content [2], black goats adapted well to the ML diet in the present study, which led to an increase in DMI. Especially in the black goats of ML15 and ML20 groups, the DMI increased in the first week of the feeding trial, and this advantage persisted until the final week of the feeding trial. It was reported that the nutrients digestibility linearly increased with the level of ML supplemented in the diet of beef cattle [15] and black goats [8]. The apparent digestibility of nutrients is closely related to the growth rate of animals. Thus, the increased DMI and nutrients digestibility in ML-supplemented black goats might be responsible for the increases in final BW and ADG.

ML have been reported to enhance the immunity and antioxidant capacity of animals [16,17]. Among the bioactive components in ML, polysaccharides are the primary active substances with various bioactive functions, such as antioxidant and immunomodulatory activities [3]. These polysaccharides demonstrate antioxidant effects both *in vitro* and *in vivo*, as evidenced by increased 1,1-diphenyl-2-picrylhydrazyl scavenging, hydroxyl radical scavenging, superoxide radical scavenging, 2,2′-azinobis-(3-ethyl-benzothiazolin-6-sulfonic acid) radical cation scavenging, and Fe^2+^ chelating activities [18], as well as the reduced levels of 8-hydroxy-2-deoxyguanosine and MDA in the livers of diabetic rats [19]. The immunomodulatory activity of ML polysaccharides is reflected by increased concentrations of immunoglobulins and inflammatory cytokines in the serum of chicks [16] or in the intestine of mice [20]. Moreover, ML contain approximately 1% to 3% (of DM) flavonoids, which are known for their significant antioxidant properties [21]. For instance, flavonoids derived from ML protected the equine skeletal muscle satellite cells from H_2_O_2_-induced oxidative damage by increasing the activities of SOD and T-AOC, and decreasing the production of MDA in H_2_O_2_-induced cells [22]. In addition to polysaccharides and flavonoids, phenolic acids extracted from ML are also reported to possess anti-inflammatory and antioxidant properties [23]. The T-AOC, SOD, and CAT are endogenous antioxidant enzymes [24], while MDA is generated by reactive oxygen species caused by oxidative stress [25]. Serum immunoglobulins act as antibodies to prevent infections [26]. Therefore, the increased levels of serum T-AOC, SOD, CAT, and IgG, along with the decreased serum MDA in ML15 goats, suggest an enhanced antioxidant and immunomodulatory activity in goats being fed with ML.

The compositions of fatty acids and amino acids are the key factors affecting the quality, nutritional value, and flavor of meat [27,28]. It was reported that a TMR diet containing 40% ML could increase total fatty acids, unsaturated fatty acids, MUFA, and PUFA, as well as total amino acid, EAA, NEAA, and flavor amino acids, while decrease SFA in the meat of black goats [8]. Although not as pronounced, similar changes were observed in the present study. Stearic acid (C18:0) is one of the major substances that cause the off-flavor in lamb [29]. In the present study, the decrease of SFA in the meat of ML20 goats was mainly due to the decrease of C18:0, which was suggested to reduce the off-flavor of mutton. Tyr is one of the aromatic amino acids that serve as precursors to the volatile flavors in meat [30]. The higher levels of Tyr in the meat of goats fed by ML suggest a potential increase in volatile flavor. Although ML was observed to alter meat fatty acid and amino acid profiles [7,8], the mechanism was not clear. It was reported that the ML polyphenols could alter lipid metabolism and change the fatty acid composition of adipose tissue in mice [31]. The ML extract is also reported to modify the fatty acid profile in female broilers through adenosine-activated protein kinase/sterol regulatory element binding protein-1c/acetyl-CoA carboxylase signaling pathway [32]. In addition, both the ML polyphenols and water extract were reported to modulate the gut microbiota in the rat and mice models, such as *Lactobacillus*, *Clostridiales*, *Lachnospiraceae*, *Blautia*, *Oscillibacter*, and *Roseburia* [33,34]. These changed microbiota were associated with lipid metabolism or amino acid metabolism [33,34]. Thus, the bioactive compounds of ML might be responsible for the changes in meat fatty acid and amino acid profiles through alteration in gut microbiota or signaling pathways, while the exact compound and mechanism need further confirmation.

## CONCLUSION

Our results indicate that ML supplementation in diets could improve the growth performance of Xiangdong black goats. By feeding with ML containing diets, the antioxidant and immunomodulatory activities of the goats improved, as evidenced by increased levels of serum T-AOC and IgG. The concentrations of SFA and Try decreased and tended to increase with ML supplementation, respectively, thereby enhancing the flavor of the meat. According to our findings, a diet supplemented with 15% to 20% ML is suitable for Xiangdong black goats. Specifically, a diet containing 15% ML showed better effects in promoting antioxidant and immunomodulatory activities, while a diet with 20% ML was more effective in enhancing meat flavor. The present study only included 5 goats in each group. Further studies with a larger sample size are necessary to determine whether a higher ML content could yield even greater benefits for Xiangdong black goats. Additionally, the specific ML extract compounds and their mechanisms in promoting growth performance, antioxidant properties, and immunomodulatory activities, as well as their effects on altering meat fatty acids and amino acid profiles, warrant further investigation.

## Figures and Tables

**Table 1:** Ingredients and nutrient level of diets fed to Xiangdong black goat

Items	Group^1)^				

	Con	ML5	ML10	ML15	ML20
Ingredients (air-dry basis, %)					
Cracked corn grain	35.5	35.6	35.0	35.5	34.5
Soybean meal	12.5	10.4	8.0	6.0	3.5
Wheat bran	11.0	10.0	10.5	9.0	11.0
Rice straw	39.0	37.0	34.5	32.5	29
Mulberry leaf powder	0	5.0	10.0	15.0	20.0
NaHCO_3_	0.5	0.5	0.5	0.5	0.5
Salt	0.5	0.5	0.5	0.5	0.5
Premix^2)^	1.0	1.0	1.0	1.0	1.0
Chemical composition (%)					
ME (MJ/kg)	7.2	7.2	7.2	7.2	7.2
DM	85.8	85.8	85.7	85.9	86.0
CP	11.8	11.9	12.0	11.9	11.9
EE	1.1	1.0	1.0	1.2	1.2
NDF	35.1	34.3	33.8	33.0	32.4
ADF	19.0	18.8	18.2	17.8	17.4
Ash	12.1	12.0	12.0	11.9	12.1
Calcium	0.56	0.50	0.48	0.53	0.57
Phosphorus	0.31	0.31	0.32	0.33	0.33

ME, metabolism energy; DM, dry matter; CP, crude protein; EE, ether extract; NDF, neutral detergent fiber; ADF, acid detergent fiber.

1) Con, control; ML, mulberry leaf.

2) The premix containing 200,000 IU/kg vitamin A, 30,000 IU/kg vitamin D3, 2000 IU/kg vitamin E, 1.5 g/kg Fe, 0.3 g/kg Cu, 2 g/kg Mn, 3 g/kg Zn, 60 mg/kg I, 20 mg/kg Co, and 30 mg/kg Se.

**Table 2:** Effects of mulberry leaf supplementation on the growth performance of Xiangdong black goat

Items	Groups^1)^					SEM	p-value	
	
	Con	ML5	ML10	ML15	ML20		Linear	Quadratic
IBW (kg)	12.7	13.0	12.7	13.2	14.2	0.43	0.323	0.526
FBW (kg)	23.3	23.5	24.1	26.9	29.5	0.97	0.014	0.033
ADG (g/d)	114.3^a^	113.8^a^	123.7^ab^	148.6^ab^	166.1^b^	7.44	0.003	0.009
IDMI (kg)	0.87^ab^	0.86^a^	0.89^b^	0.94^c^	1.11^d^	0.017	<0.001	<0.001
FDMI (kg)	1.08^b^	0.96^a^	1.06^b^	1.24^c^	1.32^d^	0.023	<0.001	<0.001
ADG/DMI	0.12	0.12	0.12	0.13	0.14	0.005	0.258	0.521

SEM, standard error of the mean; IBW, initial body weight; FBW, final body weight; ADG, average daily gain; IDMI, initial dry matter intake; FDMI, final dry matter intake.

1) Con, control; ML, mulberry leaf.

a–d Means followed by different superscripts are significantly different (p<0.05).

**Table 3:** Effects of mulberry leaf supplementation on the serum variables of Xiangdong black goat

Items	Groups^1)^					SEM	p-value	
	
	Con	ML5	ML10	ML15	ML20		Linear	Quadratic
GLU (mmol/L)	2.7	3.2	2.9	3.3	3.8	0.17	0.063	0.165
TP (g/L)	84.5	64.7	81.7	76.6	80.2	3.51	0.904	0.730
BUN (mmol/L)	3.2	3.3	3.4	3.2	2.9	0.09	0.370	0.232
CHOL (mmol/L)	4.0	3.5	4.0	3.2	4.0	0.17	0.867	0.664
TG (mmol/L)	1.2	1.2	1.1	1.2	1.1	0.05	0.792	0.966
HDL (mmol/L)	1.5	1.3	1.3	1.7	1.5	0.05	0.263	0.321
LDL (mmol/L)	2.0	2.4	2.5	2.0	1.9	0.10	0.464	0.113
VLDL (mmol/L)	0.41	0.41	0.40	0.40	0.46	0.010	0.222	0.129
IgA (g/L)	3.7	3.6	3.6	3.8	3.6	0.11	0.937	0.997
IgG (g/L)	10.7^a^	11.0^ab^	13.4^b^	13.4^b^	11.2^ab^	0.42	0.256	0.049
IgM (g/L)	1.4	1.2	1.4	1.6	1.4	0.06	0.306	0.603
C3 (g/L)	1.3	1.1	1.3	1.4	1.3	0.06	0.447	0.751
C4 (g/L)	0.25	0.24	0.27	0.27	0.25	0.010	0.691	0.724

SEM, standard error of the mean; GLU, glucose; TP, total protein; BUN, blood urea nitrogen; CHOL, cholesterol; TG, triglycerides; HDL, high-density lipoprotein; LDLC, low-density lipoprotein; VLDL, very low-density lipoprotein; IgA, immunoglobulin A; IgG, immunoglobulin G; IgM, immunoglobulin M; C3, complement 3; C4, complement 4.

1) Con, control; ML, mulberry leaf.

a,b Means followed by different superscripts are significantly different (p<0.05).

**Table 4:** Effects of mulberry leaf supplementation on the antioxidant properties of Xiangdong black goat

Items	Groups^1)^					SEM	p-value	
	
	Con	ML5	ML10	ML15	ML20		Linear	Quadratic
T-AOC (U/mL)	66.1^a^	74.4^ab^	74.0^ab^	87.5^b^	81.3^ab^	2.75	0.018	0.051
SOD (U/mL)	454.5^a^	486.3^ab^	539.8^ab^	578.7^b^	500.6^ab^	17.11	0.131	0.070
CAT (U/mL)	46.7^a^	58.9^ab^	56.1^ab^	64.8^b^	54.4^ab^	2.53	0.249	0.138
MDA (nmol/mL)	127.8^ab^	148.5^b^	117.1^ab^	86.2^a^	113.4^ab^	7.91	0.105	0.278

SEM, standard error of the mean; T-AOC, total antioxidant capacity; SOD, superoxide dismutase; CAT, catalase; MDA, malondialdehyde.

1) Con, control; ML, mulberry leaf.

a,b Means followed by different superscripts are significantly different (p<0.05).

**Table 5:** Effects of mulberry leaf supplementation on the amino acids composition in the muscle of Xiangdong black goat (mg/100 g)

Items	Groups^1)^					SEM	p-value	
	
	Con	ML5	ML10	ML15	ML20		Linear	Quadratic
Lys	61.4	63.1	65.6	63.7	65.2	1.22	0.364	0.609
Phe	32.6	33.8	34.9	33.6	35.2	0.65	0.304	0.581
Met	21.9	23.3	27.6	26.4	25.0	0.99	0.195	0.168
Thr	34.6	35.4	37.0	35.8	36.6	0.67	0.369	0.611
Val	46.6	48.5	49.9	48.2	50.0	0.90	0.328	0.580
Leu	59.9	62.3	64.9	63.1	64.2	1.26	0.315	0.505
Ile	38.8	40.3	42.5	41.4	41.7	0.83	0.254	0.387
His	21.7	23.9	24.1	23.8	21.1	0.69	0.790	0.212
Asp	68.7	71.1	74.0	71.4	73.4	1.36	0.343	0.565
Glu	117.9	122.3	129.0	124.0	126.8	2.46	0.281	0.442
Gly	36.1	37.4	36.9	32.1	40.0	1.39	0.804	0.744
Ala	45.3	46.8	48.5	45.2	49.1	0.93	0.380	0.691
Pro	38.3	39.2	39.8	35.7	42.5	1.17	0.558	0.663
Ser	25.2	25.5	27.4	26.0	26.8	0.54	0.359	0.598
Tyr	42.7	44.3	48.5	47.1	47.6	1.04	0.087	0.160
Cys	8.6	9.2	9.5	9.4	9.5	0.25	0.264	0.453
Arg	59.2	60.5	63.5	59.8	63.5	1.18	0.363	0.663
EAA	317.7	330.7	346.4	335.9	339.0	6.59	0.321	0.447
NEAA	442.1	456.3	477.0	450.6	479.3	9.06	0.300	0.582
TAA	759.7	787.0	823.4	786.6	818.3	15.24	0.296	0.525
EAA/NEAA (%)	71.9	72.7	72.7	74.4	70.6	0.63	0.876	0.385
EAA/TAA (%)	41.8	42.1	42.1	42.7	41.4	0.21	0.866	0.390
NEAA/TAA (%)	58.2	57.9	57.9	57.3	58.6	0.21	0.866	0.390
LAA	83.3	86.5	93.2	90.1	90.2	1.94	0.217	0.296
BCAA	145.4	151.1	157.3	152.6	155.9	2.97	0.298	0.492
FAA	237.1	245.1	257.4	246.9	254.5	4.82	0.300	0.507
DAA	369.9	382.4	400.3	379.6	400.4	7.55	0.294	0.557

SEM, standard error of the mean; EAA, essential amino acids (sum of Lys, Phe, Met, Thr, Ile, Leu, Val, and His); NEAA, non-essential amino acid (sum of Asp, Ser, Glu, Gly, Ala, Cys, Tyr, Pro, and Arg); TAA, total amino acids; LAA, limited amino acids (sum of Lys and Met); BCAA, branched-chain amino acids (sum of Val, Ile, and Leu); FAA, functional amino acids (sum of Glu, Leu, and Arg); DAA, flavor amino acids (sum of Asp, Glu, Gly, Ala, Arg, and Tyr).

1) Con, control; ML, mulberry leaf.

**Table 6:** Effects of mulberry leaf supplementation on the fatty acid composition in the muscle of Xiangdong black goat (g/100 g of total fatty acid methyl esters)

Items	Groups^1)^					SEM	p-value	
	
	Con	ML5	ML10	ML15	ML20		Linear	Quadratic
C14:0	1.5	1.6	1.6	1.7	1.5	0.09	0.848	0.897
C16:0	20.3	21.7	20.8	20.5	20.8	0.35	0.936	0.893
C16:1	2.1	2.2	2.2	2.4	2.4	0.10	0.325	0.624
C17:0	2.2	2.1	2.1	2.0	2.3	0.10	0.972	0.726
C18:0	17.6^a^	16.8^ab^	17.2^ab^	15.8^ab^	14.7^b^	0.64	0.146	0.325
C18:1 n9t	2.5	3.3	1.9	1.8	2.6	0.20	0.440	0.584
C18:1 n9c	47.5	44.6	45.5	47.3	46.6	0.95	0.894	0.810
C18:2 n6c	4.1	5.1	4.4	4.5	6.1	0.36	0.183	0.337
C18:3 n3	0.17	0.26	0.29	0.17	0.17	0.022	0.604	0.205
C18:3 n6	0.04	0.06	0.06	0.05	0.06	0.006	0.629	0.803
C20:0	0.07	0.07	0.08	0.07	0.07	0.007	0.886	0.759
C20:1	0.15	0.16	0.16	0.14	0.16	0.007	0.908	0.992
C20:3 n6	0.13	0.17	0.28	0.15	0.20	0.031	0.633	0.649
C20:4 n6	1.6	1.7	3.3	1.6	2.2	0.39	0.706	0.740
C22:6 n3	0.08	0.15	0.25	0.10	0.13	0.032	0.832	0.461
SFA	41.7	42.3	41.8	40.1	39.4	0.49	0.046	0.091
MUFA	52.2	50.2	49.7	51.0	51.7	1.02	0.974	0.723
PUFA	6.1	7.5	8.5	6.6	8.8	0.73	0.390	0.689

SEM, standard error of the mean; SFA, saturated fatty acids (sum of C14:0, C16:0, C17:0, C18:0, C20:0); MUFA, monounsaturated fatty acids (sum of C16:1, C18:1, C22:^1)^; PUFA, polyunsaturated fatty acids (sum of C18:2, C18:3, C20:2, C20:3, C20:4, C22:6).

1) Con, control; ML, mulberry leaf.

a,b Means followed by different superscripts are significantly different (p<0.05).
